# PD89 Guidance For Designing Pivotal Studies Of High-Risk Medical Devices By International Regulatory Authorities: A Systematic Review

**DOI:** 10.1017/S0266462324003362

**Published:** 2025-01-07

**Authors:** Petra Schnell-Inderst, Ursula Rochau, Alan G. Fraser, Uwe Siebert

## Abstract

**Introduction:**

Health technology assessment of medical devices often faces the challenge that approval studies have an insufficient study design. The European Medical Device Regulation (MDR), which has been in force since May 2021, places stricter requirements on clinical studies for high-risk medical devices. One important way of implementing the regulation is through guidance documents from the Medical Device Coordination Group (MDCG).

**Methods:**

Our objective was to systematically identify and analyze recommendations for the design of pivotal studies of high-risk therapeutic medical devices from the MDCG and other regulatory authorities of high-income countries. We systematically searched the websites of regulatory authorities and included both cross-device and device-specific recommendations on clinical trial design for cardiovascular, orthopedic, and diabetes related high-risk medical devices. We extracted and compared the recommendations according to the following seven topics: definitions of study types and level of evidence; need for clinical investigations; appropriate choice of study methodology; general aspects; statistical methods; consideration of context and learning curve; and reporting.

**Results:**

We included 30 documents from regulatory authorities. Eight documents from the European Union (EU) focused mainly on procedural issues and reporting. Guidance on appropriate levels of evidence and choice of study methodology for design and analysis of clinical investigations was missing. Eleven guidance documents were from the United States Food and Drug Administration, which provided detailed recommendations on almost all topics regarding study design as well as new methods. The remaining documents originated from four other countries and the International Medical Device Regulators Forum. A document from Australia included device-specific recommendations but hardly any general recommendations on clinical trials.

**Conclusions:**

The recommendations published to date in the EU are insufficient to support stakeholders in choosing or assessing appropriate study designs to implement the evidence requirements of the European MDR. Detailed guidance from the MDCG is needed to create a common understanding between manufacturers, notified bodies, MDR expert panels, and competent authorities regarding what sufficiency of clinical data means.

